# Demineralization and sectioning of human kidney stones: A molecular investigation revealing the spatial heterogeneity of the stone matrix

**DOI:** 10.14814/phy2.14658

**Published:** 2021-01-06

**Authors:** Victor Hugo Canela, Sharon B. Bledsoe, James E. Lingeman, Glenn Gerber, Elaine M. Worcester, Tarek M. El‐Achkar, James C. Williams

**Affiliations:** ^1^ Department of Anatomy, Cell Biology & Physiology Indiana University School of Medicine Indianapolis IN USA; ^2^ Department of Urology Indiana University School of Medicine Indianapolis IN USA; ^3^ Section of Nephrology Department of Medicine University of Chicago Chicago IL USA; ^4^ Division of Nephrology Department of Medicine Indiana University and Roudebush Indianapolis Veterans Affairs Medical Center Indianapolis IN USA

**Keywords:** calculi, nephrolithiasis, stone matrix, stones

## Abstract

The molecular mechanisms by which kidney stones grow are largely unknown. Organic molecules from the urine combine with mineral crystals to form stones, but analysis of the stone matrix has revealed over a thousand different proteins, with no clues as to which are important for stone growth. Molecules that are present in every layer of a stone would be candidates for having an essential function, and thus the analysis of the stone matrix at a microscopic level is necessary. For this purpose, kidney stones were demineralized, sectioned, stained, and imaged by microscopy, using micro CT for precise orientation. Histological staining demonstrated heterogeneity in the density of adjacent layers within stones. Additional results also showed brilliant and unique autofluorescence patterns in decalcified nephroliths, indicating heterogeneous organic composition in adjacent layers. Regions of calcium oxalate (CaOx) stones were dissected using laser microdissection (LMD) for protein analysis. LMD of broad regions of demineralized CaOx stone sections yielded the same proteins as those found in different specimens of pulverized CaOx stones. These innovative methodologies will allow spatial mapping of protein composition within the heterogeneous stone matrix. Proteins that consistently coincide spatially with mineral deposition would be candidates for molecules essential for stone growth. This kind of analysis will be required to assess which of the thousand proteins in the stone matrix may be fundamental for stone growth.

## Graphical abstract

Our innovative methodology describes a novel laboratory method to study human kidney stones, whereby the stone matrix is microscopically analyzed to reveal its molecular spatial heterogeneity. It is our intent that the data presented in this manuscript will foster future molecular interrogations into the stone matrix for a better understanding of the underlying mechanisms contributing to renal stone formation.

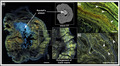

## 1 INTRODUCTION

Kidney stone prevalence continues to increase, reaching as high as 14.8%, and more than half of affected individuals will have a stone recurrence within 10 years of their first episode (Khan et al., 2016). Health costs in the United States for kidney stone treatment and management currently exceed $10 billion annually (Scales et al., 2016). Risk factors for nephrolithiasis—including diabetes, hypertension, and obesity—are also on the rise.

Surprisingly, very little is known about the precise mechanisms of kidney stone growth. Meticulous efforts have been made to grow calcium oxalate (CaOx) stones in vitro, including control of composition and temperature over periods of weeks (Chow et al., 2004a, 2004b), but the “stones” produced consisted of fragile material that disintegrated when touched (Ananth et al., 2002).

The failure of inorganic solutions to produce a stone with the hardness typical of nephroliths points to some role of the organic matrix in stone formation. It has been suggested that the deposition of the stone matrix is primary in forming a stone, with crystal deposition within the matrix being a secondary event (Boyce, 1968; Boyce & Sulkin, 1956). Ultrastructural analysis of CaOx stones suggests that each mineral crystal is surrounded by a layer of matrix (Evan et al., 2007; Khan & Hackett, 1993).

Proteomic analysis of the CaOx kidney stone matrix has revealed the presence of over a thousand different proteins (Witzmann et al., 2016). How can one sort through so many possible molecules to see which ones might be fundamental for stone growth? One approach would be to find out which proteins are present in every mineral layer, hypothesizing that if a protein is fundamental to stone growth, it would necessarily always be present. The present study utilized the micro CT orientation of stones during demineralization, embedment, and sectioning, and subsequent analysis of matrix layers by fluorescence microscopy, immunohistochemistry, and regional proteomics using laser microdissection (LMD). This novel approach shows promise of being capable of distinguishing proteins that are universally present from those that occur in only some mineral layers and thereby could identify proteins that would be candidates for possessing essential function in stone growth.

## 2 METHODS

### 2.1 Stone specimens

Stones were collected during endoscopic procedures as part of an IRB‐approved study (IU IRB #1010002261) or were received as cast‐offs from a local stone analysis facility (Beck Analytical Services). These stones came to us without any other patient or clinical identifiers. Stones were photographed, dried, and scanned using micro CT (Skyscan 1172 Micro CT system; Bruker‐Micro CT) with final voxel sizes of 2–8 µm. Kidney stones reported in this paper include only those for which CaOx monohydrate (COM, crystal name: whewellite) was the majority mineral. Mineral identifications were confirmed using infrared spectroscopic methods (Williams et al., 2010).

### 2.2 Kidney stone preparation and demineralization

**FIGURE 1:**
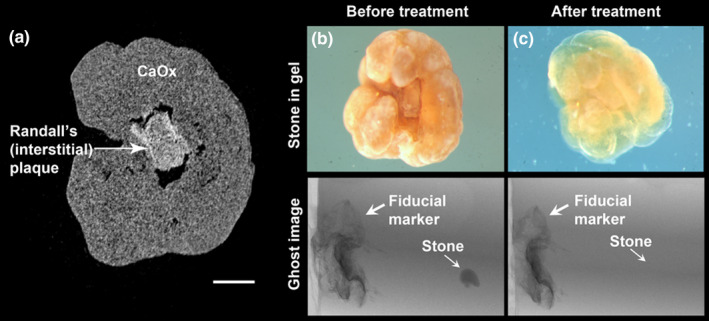
Micro CT ensures proper orientation and positioning of Randall's plaque stone for histology and accurately assesses mineral loss. (a) A micro CT slice showing the appearance of a stone attached to Randall's (interstitial) plaque. Scale bar = 250 µm. CaOx: calcium oxalate (which, in this case, was pure CaOx monohydrate, COM). Photographic images (upper portions of panels b and c) show the stone's overall structure within a gel before and after demineralization. The lower panels of b and c show the before and after micro CT “ghost” images of a CaOx stone on Randall's plaque undergoing decalcification, with no visibility by micro CT at the end of the process. This stone came from a patient who underwent bilateral ureteroscopy for multiple renal stones. The patient was a 78‐year‐old male, with a history of passing stones. Urine was typical for a CaOx stone former, with multiple 24‐hr collections averaging 2.9 L volume, pH 6.61, Ca of 278 mg, oxalate of 35 mg, citrate > 1,200 mg, and a supersaturation for CaOx of 4.7 and for CaP of 1.6

Kidney stones were embedded in 6%–7% Knox gelatin (Kraft Foods) or HistoGel (Thermo Fisher Scientific) and scanned by micro CT to confirm orientation. Subsequently, the gel embedded stones were immersed in a 1:1 ratio of 5% paraformaldehyde and 10% ethylenediaminetetraacetic acid, pH 7.4, at room temperature, refreshed daily. Demineralization was deemed to be complete when the stone was invisible by micro CT (Figure 1).

### 2.3 Kidney stone histology

Demineralized stones were dehydrated through a series of ethanol concentrations, infiltrated, and embedded in a 1:1 mixture of Paraplast X‐TRA (Fisher Scientific) and Peel‐Away Micro‐Cut (Polysciences Inc.). Sections were cut at 6–8 µm. Hematoxylin and eosin (H&E) and Yasue staining were used for histological analysis (Evan et al., 2003). Unstained serial sections of kidney stones were also imaged by fluorescence using a Leica TCS SP8 confocal imaging system (excitation at 405, 488, 552, and 643 nm) for native fluorescence.

### 2.4 Super‐Resolution Auto‐Fluorescence

Super‐resolution auto‐fluorescence (SRAF) imaging analyses of decalcified COM stone matrix were run in the Core Facilities of the Carl R. Woese Institute for Genomic Biology at the University of Illinois Urbana‐Champaign. Images were acquired on a Zeiss LSM 880 Airyscan SRAF system (Sivaguru et al., 2018).

### 2.5 Immunostaining

Kidney stone sections were deparaffinized and rehydrated, and antigen retrieval was performed in preheated sodium citrate buffer, pH 6.0. Subsequently, sections were incubated with a sheep polyclonal antibody against human Tamm–Horsfall Protein (THP; Millipore) and stored at room temperature overnight. THP immunoreactivity was assessed using a rabbit anti‐sheep IgG secondary antibody conjugated with horseradish peroxidase and visualized with 3,3′‐diaminobenzidine tetrahydrochloride hydrate. The sections were counterstained with hematoxylin and viewed using a Leica DM3000.

Sections of stones were mounted on membrane slides for the “move and cut” mode of a Leica LMD 6 microscope instrument (Micanovic et al., 2015).

### 2.6 Regional proteomics using LMD

Sections were deparaffinized and stained with either H&E or 1% methylene blue. Bulk regions of the stone matrix layers were dissected via LMD and collected in phosphate buffer. Extracted proteins were digested using Trypsin/Lysine C mix proteases and run in a Q Exactive Orbitrap High Field Liquid Chromatography/Mass Spectrometry system. Data were searched using MaxQuant (version 1.6.10.1) and filtered using False Discovery Rate for α = 0.01. Hierarchical clustering analyses of these data were conducted using the Perseus computational software (Tyanova et al., 2016).

## 3 RESULTS

### 3.1 Micro CT visualization secures and guides proper kidney stone orientation and demineralization

Micro CT examination ensured proper stone orientation and positioning for histological sectioning. The loss of mineral density by micro CT provided a direct measure of the adequacy of demineralization. Photographic images in Figure 1 show an example of Randall's plaque stone undergoing the process of demineralization; the stone's overall structure was maintained within the gel even after complete demineralization (Figure 1c).

### 3.2 Histological staining demonstrates matrix heterogeneity and confirms the complete decalcification of the kidney stones

**FIGURE 2:**
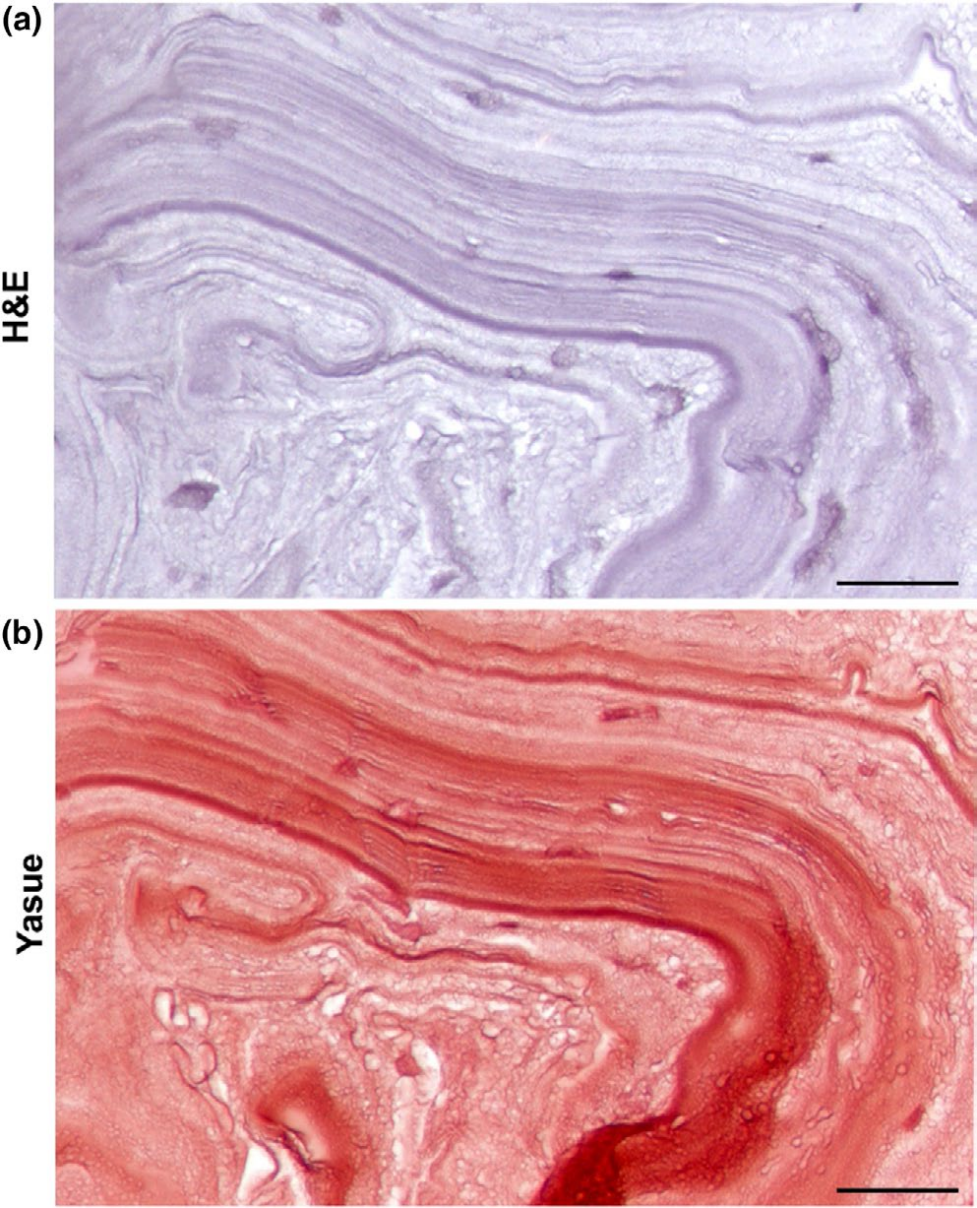
Hematoxylin and eosin (H&E) and Yasue staining show stone matrix heterogeneity. (a) H&E of a decalcified COM stone showed evidence of matrix heterogeneity. The various levels of staining intensities among the tightly packed layers suggest differences in organics throughout the matrix. (b) Yasue staining also clearly depicts matrix heterogeneity. Additionally, this staining technique verifies the complete demineralization of the stone as indicated by micro CT. Scale bars = 50 µm. These specimens came to us de‐identified

Hematoxylin and eosin and Yasue staining of kidney stone sections showed tightly packed layers in the matrix of this pure COM stone (Figure 2, H&E). Both staining methods showed heterogeneity in the density of the stainable organic matrix among different layers within the stones. The absence of staining for calcium mineral in panel B (Yasue) also verified the complete demineralization of the stone indicated by micro CT.

### 3.3 Native fluorescence of the stone matrix reveals its molecular heterogeneity by various autofluorescent signatures

**FIGURE 3:**
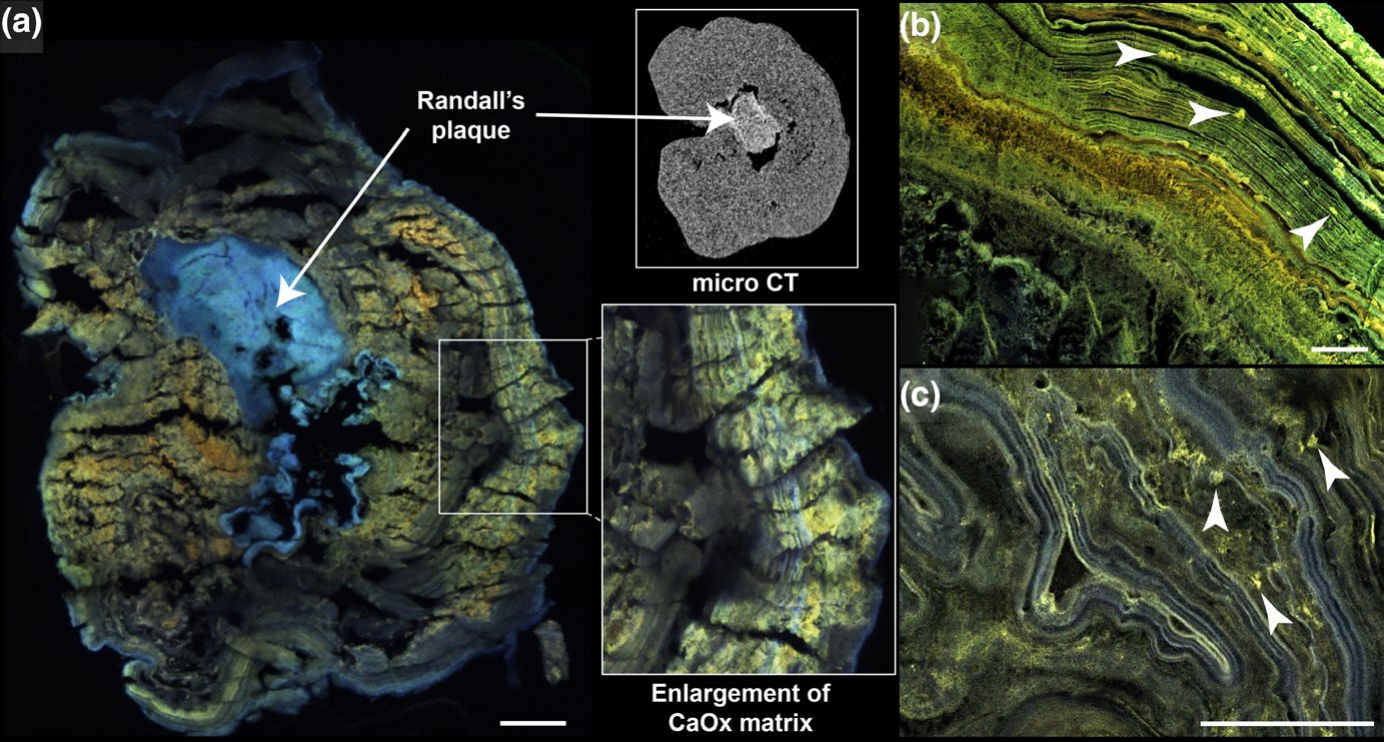
Heterogeneity of native fluorescence in stone matrices. (a) The decalcified stone (from Figure 1) displayed fluorescence in the far‐blue region in the area that used to be apatite rich (Randall's plaque). The upper right inset shows a micro CT slice of the stone prior to demineralization, in approximately the same plane as the histologic section. The lower right inset shows an enlargement of the CaOx matrix layers of differing intensities and colors. (b) The stone matrix in this panel depicts different levels of fluorescence intensities and colors from two other patient specimens (both de‐identified); additionally, microscopic organic aggregates can be appreciated (white arrowheads). (c) Fluorescent aggregates (white arrowheads) can also be seen in the matrix of this stone. Further, this stone possessed pronounced differences in fluorescence color between adjacent layers, indicating evident heterogeneity in matrix composition. Scale bars = 100 µm

Figure 3 shows three different decalcified CaOx monohydrate stones taken from three different patients, imaged for native matrix fluorescence. Figure 3a shows the same stone from Figure 1, whereby the orientation of demineralized stone sections was maintained with high fidelity. The demineralized Randall's plaque displayed a unique fluorescence pattern in the far‐blue region, as we have previously described in mineralized plaque (Winfree et al., 2019). The CaOx surrounding the plaque region showed multiple matrix layers of differing intensities and fluorescence emission spectra, suggesting molecular heterogeneity in the composition of the matrix. The specific autofluorescent signatures of each, plaque and CaOx stone overgrowth regions, describes the unique molecular makeup of the calcified papillary interstitium and the calyceal urine‐containing space. Furthermore, panels B and C show two additional CaOx stones with varied native fluorescent signatures, pointing to the molecular heterogeneity within the organic matrix of COM.

**FIGURE 4:**
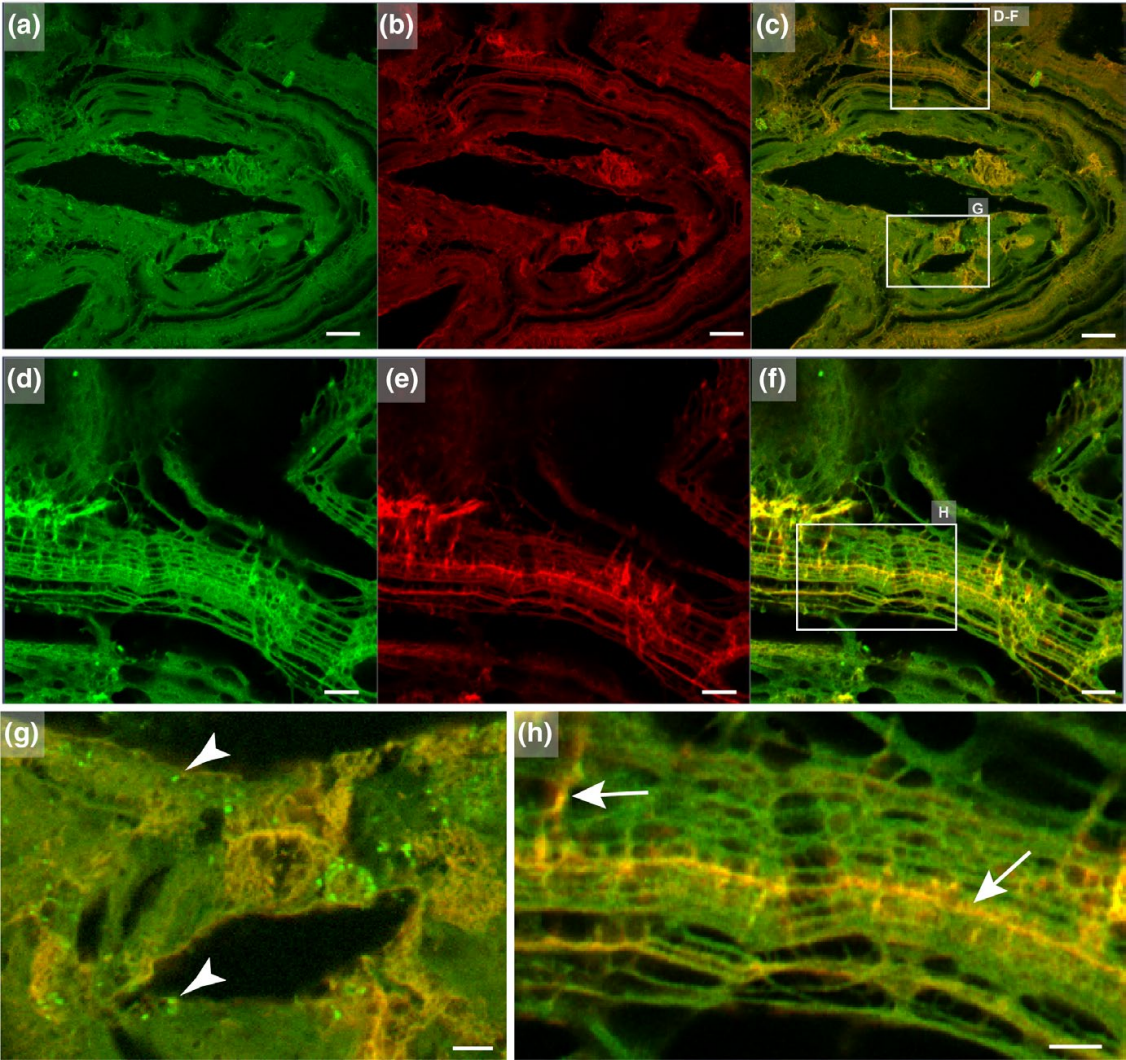
Super‐resolution auto‐fluorescence (SRAF) imaging renders the finer resolution of the heterogeneous stone matrix. (a) Low‐power SRAF image of a CaOx stone matrix (blue‐405 nm excitation, 420–480 nm emission channel pseudo‐colored green, scale bar = 20 µm). (b) Low‐power SRAF image (red‐561 nm excitation, 575–615 nm emission channel pseudo‐colored red). (c) Merged images of a and b. (d) High‐power SRAF image of d–f box in c (blue‐405 nm excitation, 420–480 nm emission channel pseudo‐colored green, scale bar = 5 µm). (e) High‐power image of box d–f box in c (red‐561 nm excitation, 575–615 nm emission channel pseudo‐colored red). (f) Merger of d and e. (g) Enlargement of box g in c with merged images of blue and red channels pseudo‐colored green and red, respectively. Arrowheads indicate organic aggregates entrapped within the stone matrix (scale bar = 5 µm). (h) Enlargement of box h in f merged images of blue and red channels pseudo‐colored green and red, respectively. Arrows indicate locations of green and red channel overlaps, indicating heterogeneity within the filamentous stone matrix (scale bar = 5 µm). Although the bulk of this specimen was composed of COM, the polygonal shapes of the empty spaces in panels a through c suggest the previous presence of the dihydrate form of CaOx (Sivaguru et al., 2018)

The examination of a sectioned, decalcified CaOx stone using SRAF imaging (Sivaguru et al., 2018) is shown in Figure 4. The filamentous nature of the matrix and its heterogeneity is further revealed by the high‐resolution SRAF optics (Figure 4d–h).

### 3.4 Immunohistochemical staining

**FIGURE 5:**
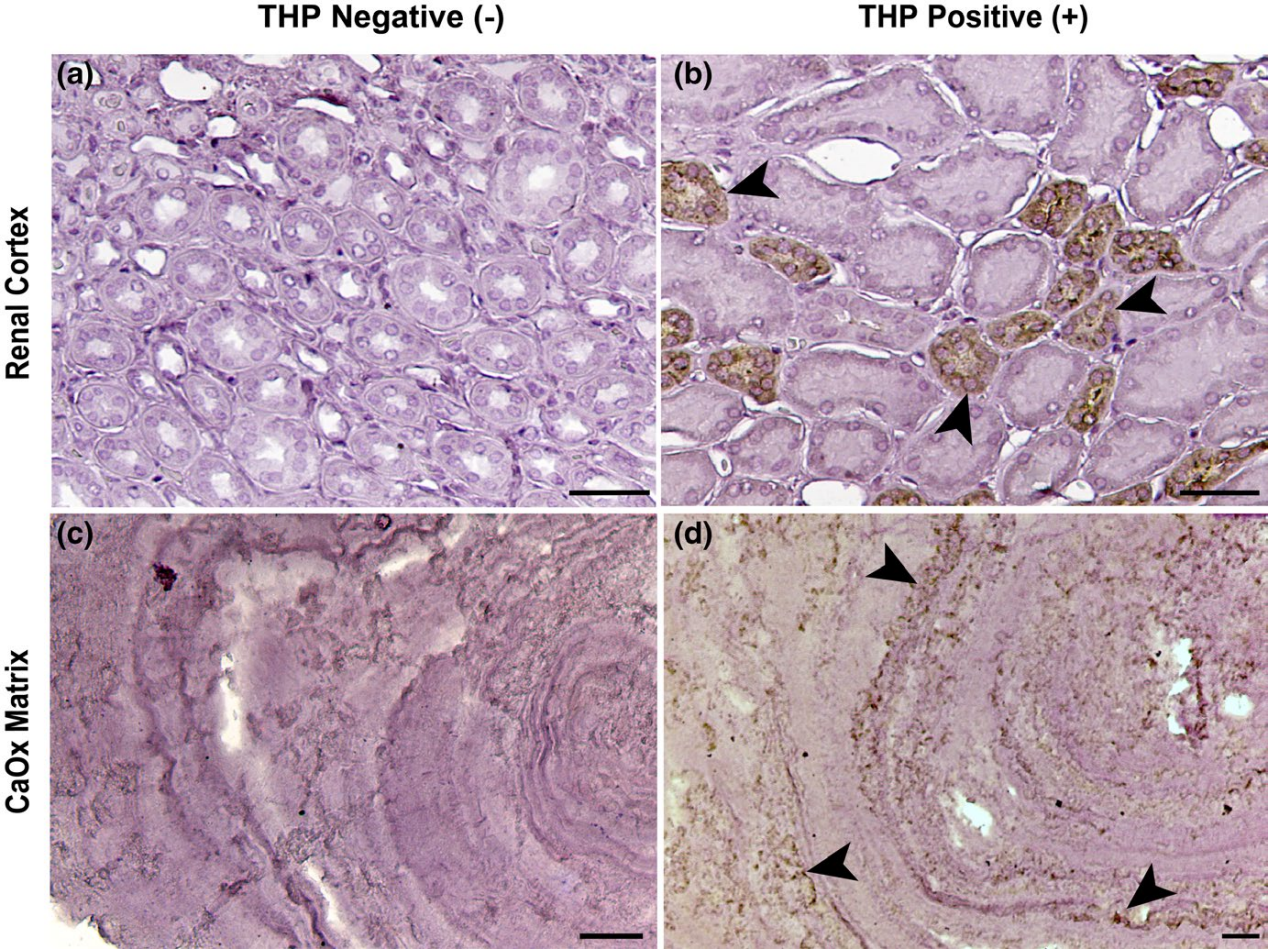
Alternating layers of Tamm–Horsfall Protein (THP) in the stone matrix is revealed by immunohistochemistry of CaOx stone matrix. No primary antibody controls (Panels a and c). Panel b shows THP reactivity (black arrowheads) in the thick ascending limbs (TAL) of a human kidney section (renal cortex). Immunohistochemistry staining of CaOx stone matrix with THP shows THP‐rich layers (black arrowheads) and THP‐absent layers in the matrix of decalcified CaOx stones (Panel d). Scale bar = 50 µm. This specimen had a mixed composition, (86% COM, 7% uric acid, and 7% apatite). The patient had undergone bilateral percutaneous stone removal, and 24‐hr urine analyses averaged 1.8 L, pH of 6.10, Ca 241 mg, oxalate 49 mg, citrate 747 mg, with supersaturations of 8.1 for CaOx and 1.38 for CaP

Kidney stone sections were also stained for Tamm–Horsfall Protein (THP), as shown in Figure 5. Our results consistently showed a pattern of alternating THP‐rich (black arrowheads) and THP‐absent layers within the stone matrix of CaOx kidney stones.

### 3.5 Regional proteomics using LMD

**FIGURE 6:**
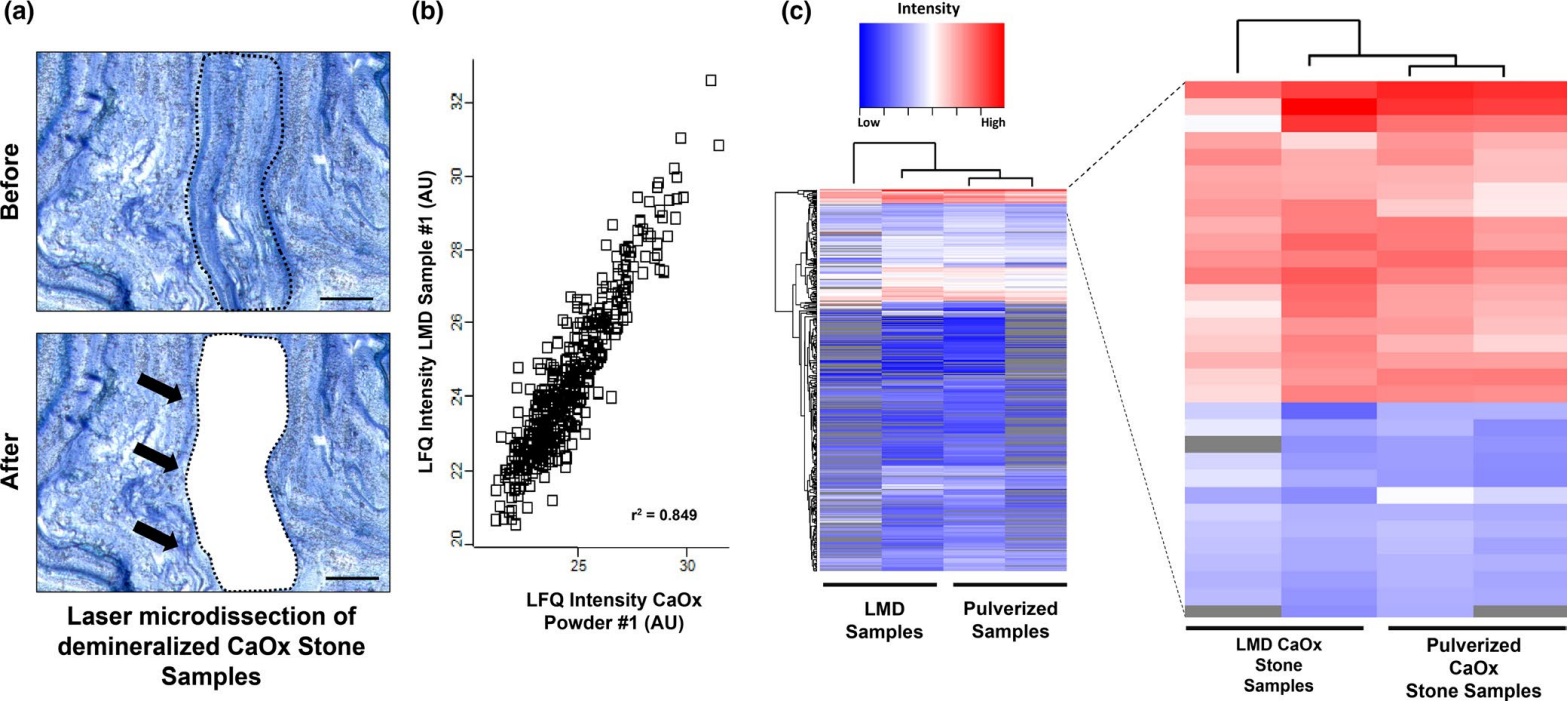
Laser microdissection (LMD) facilitates the proteomic spatial mapping of CaOx stones. (a) The process of LMD is illustrated in the before and after (black arrows) panels for protein extraction (scale bar = 50 µm). (b) Proteins identified in CaOx stone powder and LMD sections plotted against one another by their relative label‐free quantitation value (LFQ). (c) Unsupervised hierarchical clustering highlighting the most abundant proteins identified in both LMD and pulverized CaOx stone samples. AU, arbitrary units

The process of LMD of a CaOx stone specimen is illustrated in Figure 6a. The average area of LMD samples for proteomic analysis was 1.64 × 10^6^ µm^2^, and these samples yielded an average of 629 distinct proteins. When the concurrent proteomic analysis was performed with approximately 100 mg of CaOx stone powder, we identified 464 of those 629 proteins. Label‐free quantitative analysis showed a strong correlation in relative protein abundance between LMD and pulverized stone samples (Figure 6b), thereby validating the LMD‐based proteomics approach. Table 1 summarizes the twenty most abundant proteins in both LMD and pulverized CaOx kidney stone samples from the hierarchical clustering analysis from Figure 6c.

**TABLE 1:** The 20 most abundant proteins in both laser microdissection and pulverized calcium oxalate stone samples. The proteins identified are listed in order of abundance based on their label‐free quantitation value by unsupervised hierarchical clustering. Note that Tamm–Horsfall Protein (also known as uromodulin) is among the top five proteins identified

Hemoglobin subunit beta (HBB)
Hemoglobin subunit alpha (HBA2)
15‐hydroxyprostaglandin dehydrogenase [NAD^+^] (HPGD)
Fatty Acid Synthase (FAS)
Uromodulin (UMOD)
Myosin‐9 (MYH9)
Osteopontin‐D (SPP1)
Tubulin Beta chain (TUBB2C)
Heat shock protein HSP 90‐alpha (HSP90AA1)
Tubulin alpha chain
Complement C3 (C3)
Fibrinogen alpha chain (FGA)
Vitamin K‐dependent protein Z (PROZ)
Heat shock protein HSP 90‐beta (HSP90AB1)
Fibrinogen gamma chain (FGG)
Pro‐epidermal growth factor (EGF)
Mannan‐binding lectin serine protease 2 (MASP2)
Vesicular integral‐membrane protein VIP36 (LMAN2)
Coronin‐1B (CORO1B)
Beta‐1,4,galactosyltransferase 1 (B4GALT1)

## 4 DISCUSSION

The work described here illustrates an innovative method to study the kidney stone matrix and to explore its molecular (organic) content. Although investigators have previously demonstrated the feasibility of demineralizing and sectioning kidney stones to study the organic matrix (Chow et al., 2004a), we not only optimized this histological technique but extended its utility by methodically applying two novel and critical techniques: (a) micro CT was used to assess the degree of stone demineralization and, more critically, for the precise positioning and orienting of the nephroliths for histological sectioning, and (b) LMD was used for analyzing specific regions of interest of the stone matrix. Moreover, we demonstrate the advantage of these innovative methodologies by yielding significant proteomic data with laser microdissected samples and demonstrate strong proteomic correlation in agreement with pulverized stone samples. The ability to apply histochemical and proteomic techniques to kidney stones enables an in‐depth molecular interrogation that has not been demonstrated before and should aid in understanding the initiation and propagation of stone growth.

The organic matrix in CaOx stones appears to be heterogeneous. This is particularly apparent in the fluorescence images (Figures 3 and 4), indicating that the nature of the matrix can be different in adjacent layers. Of note, a mixed kidney stone with majority uric acid composition also demonstrated apparent heterogeneity by histologic staining and autofluorescence (Fig. S1). The preliminary work with immunohistochemical analysis for THP—the most abundant of the urinary proteins (Figure 5)—showed that layers of the stone matrix could be either devoid of or rich in THP‐ (Fig. S2). This suggests, then, that THP is unlikely to be a urine protein that is essential for the growth of a stone, as it was undetectable in some layers of the stone.

The number and diversity of proteins found in these preliminary studies using LMD sections are consistent with what has previously been published for larger stone specimens (Witzmann et al., 2016). The presence of such a large number of different proteins in the stone matrix is yet to be explained, but it is possible that many proteins in urine may become part of the stone matrix in a non‐specific manner. It has been proposed that certain proteins are key in the formation of a stone (Boyce, 1968), and that others may simply accumulate through long‐term exposure of the stone to urine (Witzmann et al., 2016). If this is the case, then it will be important to sort out which proteins are key, and which are simply adventitious.

The proteomic analysis of LMD sections from demineralized stones opens the possibility of analysis of specific layers within a single stone. Such work should be able to identify proteins that are present in all stone layers. Such proteins would then be candidates for focused study of their effects on the mineral deposition, perhaps using one of the modern methods that allow microscopic monitoring of mineral growth, in vitro (Singh et al., 2017).

## 5 CONCLUSIONS

Future studies of the stone matrix will aid in describing the mechanisms of mineral and organic layer deposition. Spatial mapping of the stone proteome will undoubtedly lead to a better understanding of stone initiation including renal anchoring mechanisms (i.e., Randall's plaque and/or ductal plugging). Thus, utilization and optimization of these novel techniques can begin to unlock the mysteries of CaOx mineral initiation and propagation.

## CONFLICT OF INTEREST

The authors declare no conflict of interest.

## AUTHOR CONTRIBUTIONS

VHC, TME, and JCW conceived and designed research; VHC and SBB performed experiments; JEL, EMW, and GG collected surgical samples; VHC, TME, and JCW analyzed and interpreted results of the experiments. All authors contributed to the drafting of the work and revising it for important intellectual content. All authors approved the final version of the manuscript.

## Supporting information



Supplementary Material
