# Supplementary material for: Demineralization and sectioning of human kidney stones: A molecular investigation revealing the spatial heterogeneity of the stone matrix
Source: Physiol Rep. 2021 Jan 6;9(1):e14658. doi: 10.14814/phy2.14658 (PMC7786195; doi:10.14814/phy2.14658)
Supplement: Supplementary file 1 — Supplementary Material [file PHY2-9-e14658-s001.docx]

**Supporting Information**

Supplementary figures are available: 10.6084/m9.figshare.12935186

**Figure S1. Histology of decalcified CaOx stone sections shows nodule-like structures and heterogeneity within layers.**H&E staining of stone matrix reveals the heterogeneity in layers (A) and verifies successful decalcification by Yasue staining (B). Scale bar = 50 µm.

**Figure S2. Decalcified uric acid kidney stone sections showing heterogeneity by histology staining and native fluorescence imaging.**The analyzed mixed kidney stone was composed of 74% uric acid and 26% CaOx. H&E and Yasue (**A** and **B**) staining reveals nonuniformity within layers of the uric acid stone matrix. **C.** Native fluorescence imaging shows further diversity in the structural composition of the matrix layers. Scale bar = 100 µm.

**Figure S3. Immunohistochemistry experiments reveal alternating layers of THP of the mixed CaOx and uric acid stone matrix.**No primary controls (**A**). **B.** Mixed CaOx and Uric acid matrix contain alternating layers of THP (black arrows). Scale bar = 50 µm.
